# Clinical outcomes of Preimplantation genetic testing (PGT) application in couples with chromosomal inversion, a study in the Chinese Han population

**DOI:** 10.1186/s12958-020-00635-7

**Published:** 2020-08-05

**Authors:** Yuhan Shao, Jing Li, Juanjuan Lu, Hongchang Li, Yueting Zhu, Wenjie Jiang, Junhao Yan

**Affiliations:** 1 Center for Reproductive Medicine, Cheeloo College of Medicine, Shandong University, Jinan, 250012 Shandong China; 2 National Research Center for Assisted Reproductive Technology and Reproductive Genetics, Jinan, 250012 Shandong China; 3 Key Laboratory of Reproductive Endocrinology of Ministry of Education, Shandong University, Jinan, 250012 Shandong China; 4 Shandong Provincial Clinical Medicine Research Center for Reproductive Health, Shandong University, Jinan, 250012 Shandong China

**Keywords:** Preimplantation genetics testing (PGT), Chromosomal inversion, Pregnancy outcome, Aneuploid

## Abstract

**Background:**

Chromosomal inversion was considered to have adverse effects on pregnancy outcomes through abnormal gametogenesis. The purpose of this retrospective study was to investigate whether preimplantation genetic testing (PGT) improves pregnancy outcomes for couples with chromosomal inversion.

**Methods:**

A total of 188 cycles from 165 couples with one chromosomal inversion carrier were divided into two groups: PGT (136 cycles, 125 couples) and non-PGT (52 cycles, 50 couples). Biochemical pregnancy, clinical pregnancy, ongoing pregnancy, miscarriage and live birth rates of their first transfer cycles, as well as cumulative live birth rates of each cycle and euploidy rates, were analyzed.

**Results:**

There were no statistically significant differences in the pregnancy outcomes between the two groups. The euploidy rate of pericentric inversion carriers was not higher than that of paracentric inversion carriers in PGT group (60.71% vs 50.54%, *P* = 0.073). Similarly, the euploid rate of male carriers was not higher than that of female carriers (61.2% vs 56.1%, *P* = 0.256).

**Conclusions:**

Due to limitation of retrospective study and small sample size, our current data showed that PGT cannot provide prominent benefits for inversion carriers in the Chinese Han population. Further prospective randomized controlled trials are needed to evaluate the effects of PGT.

## Introduction

Chromosomal inversion is a common structural rearrangement of chromosomes where the chromosome is cleaved into three parts by two breakages. The middle fragment undergoes a 180° rotation and reinserts itself into its original location without loss of genetic material detectable through karyotyping. Chromosomal inversion is classified into pericentric inversions and paracentric inversions. In pericentric inversions, two breakages are located in the p and q arms, respectively, and the inverted segment contains the centromere. In contrast, in paracentric inversions, two breakages are located in the same arm of a chromosome and the inverted segment does not contain the centromere [1]. It’s estimated that the frequency of pericentric and paracentric inversion in the general population is 1–2% and 0.1–0.5%, respectively [2, 3]. Most inversion carriers show no phenotypic abnormalities but are likely to produce unbalanced gametes that might cause infertility, miscarriage, stillbirth and an abnormal karyotype in the offspring. The risk for pericentric inversions of producing unbalanced progeny is about 10% for female carriers and 5% for male carriers [4].

It’s generally acknowledged that the inversion loop is formed during synapsis between the inverted chromosome and its homologous counterpart during meiosis. The unbalanced gametes are produced when there is an odd number of cross-overs within the inversion loop [5]. For inversion carriers, the risk of producing unbalanced gametes depends on the occurrence of recombination within the inverted segment, and homologous synapsis is necessary for recombination events. When homosynapsis does not take place, the occurrence of crossing-over is inhibited and no unbalanced recombinant gametes are produced [6]. When recombination occurs, unbalanced recombinant chromatids produce gametes: duplications or deletions from pericentric inversion carriers; dicentric or acentric fragments (involving duplications or deletions) from paracentric inversion carriers [7]. Theoretically, four different kinds of gametes are generated from a inversion carrier: the completely normal gamete; a balanced gamete; a deletion distal to the inverted part on p arm together with a duplication distal to the inverted part on q arm and, finally, a duplication distal to the inverted part on p arm together with a deletion distal to the inverted part on q arm. Due to meiotic challenges, inversion carriers are faced with risk of gametes production failure and/or unbalanced gametes that can lead to subfertility. Homologous sections are aligned by inverison loop’s twisting and folding the inverted segment. When the inverted segment is so long that it involves most of the chromosome length, synapsis of the inversion with its homologous noninverted segment prevails over pairing with the distal segments. In these circumstances, the distal segments remain unpaired and no inversion loop is formed [6]. Inversion carriers with small distal segments of the inverted chromosome are more likely to give birth to chromosomally unbalanced or phenotypically abnormal offspring. Accordingly, inversion heterozygotes in which one or both distal segments are large have a risk for producing gametes and recombinants with considerable genetic imbalance, leading to implantation failure or pregnancy loss [7]. Besides, there are other factors such as the location of breakpoints that are likely to affect the chance of inversion loop formation as well [6].

The chromosomes of embryos fertilized by abnormal gametes can result in a partial monosomy or a trisomy represented as congenital defects [8]. The frequency of recombination within the gametes is related to parameters such as the involved chromosome, the location of breakages, the size of the inverted segment and its proportion in relation to the size of the whole chromosome [9]. Previous studies proposed that the chance of recombination increased with the size of the inverted segment accounting for over 30% of the whole chromosome. Moreover, there seems to be a high risk of producing aneuploid embryos when the size of the inverted segment is at least 100 Mb or over 50% [5, 6]. PGT (preimplantation genetic testing) avoids transplanting unbalanced embryos by detecting abnormal embryonic chromosomal configurations before embryo transplantation and therefore this procedure might reduce the risk of subsequent implantation failure and miscarriage. However, there is not enough evidence to prove that PGT is essential for inversion carriers to improve pregnancy outcomes in that group.

During the past few years, most researchers and clinicians have considered inversion of chromosome number 9 to be a polymorphism involving only heterochromatin DNA. However, this is currently becoming controversial since a few researchers reported that it may be associated with infertility, recurrent pregnancy loss, stillbirth and other abnormal outcomes, although the available evidence is scarce [10]. Considering its high incidence and potential effects on pregnancy outcomes, here we discuss pregnancy outcomes after PGT of chromosomal inversion carriers except possible inversions on chromosome 9.

## Material and methods

### Patients

Retrospective analysis on the pregnancy outcomes of chromosomal inversion was performed on carriers who had undergone in vitro fertilization (IVF) or IVF in combination with intracytoplasmic sperm injection (ICSI) and PGT, in the Center for Reproductive Medicine of Shandong University. A total of 188 cycles were analyzed in our study from April 2012 to April 2018. These cycles were divided into two groups depending on whether they chose PGT (PGT group, 136 cycles) or not (control group, 52 cycles, IVF/ICSI). In the PGT group, indications of ART in 106 cycles were just chromosomal inversion and the remaining 30 cycles were conducted because of advanced maternal age or previous repeated miscarriages besides inversion. Clinical characteristics and pregnancy outcomes of these two groups were compared. Furthermore, patients in the PGT group were divided into four subgroups according to the type of inversion (pericentric inversion, paracentric inversion) and the gender of inversion carriers (female carrier, male carrier). Finally, the aneuploidy rates were compared between the four subgroups.

### Inclusion criteria

One person in a couple carried chromosomal inversion, and the other possessed completely normal or polymorphic karyotype.

The age of involved women ranged from 21 to 45 years old and the dates of assisted reproductive technology (ART) ranged from April, 2012 to April, 2018.

At least one morphological high-quality blastocyst was acquired.

All transfers of PGT group were cryopreserved blastocyst transfers while transfers of control group include cryopreserved and fresh blastocyst transfers.

### Methods

#### Controlled ovarian stimulation (COH)

COH protocols are chosen on the evaluation of ovarian reserve including age, anti-Müllerian hormone (AMH), basal hormone levels and antral follicle count (AFC). The long-term protocol of ovulatory induction is most frequently used, and it is mainly applied to young women with good ovarian reserve. The short-term protocol is used in women less than 40 years old whose ovarian reserve is not satisfying enough. Women with poor response to the long-term protocol or with polycystic ovarian syndrome (PCOS) can be induced by the GnRH-antagonist protocol. The ultra-long protocol is mainly suitable for female patients with endometriosis.

#### Oocyte retrieval

Human chorionic gonadotropin (hCG) was injected when at least one follicle with a diameter over 18 mm was present. Oocytes were retrieved 36 h later.

#### In vitro fertilization

IVF or IVF/ICSI was chosen according to the sperm parameters on the day of oocyte retrieval in the control group. PGT was done in IVF/ICSI cycles to eliminate the effect of sperm and guarantee the accuracy of biopsy. In our center, the indication of IVF/ICSI includes: 1) Sperm concentration < 5 X106 /ml; 2) Sperm motility< 10%; 3) Rate of sperm with normal morphology< 1%.

#### Biopsy

Firstly, blastocysts were graded according to the Gardner blastocyst morphologic scoring system [11]. Approximately 4–6 trophoblast cells from high quality blastocysts were biopsied 5–6 days after fertilization by an experienced embryologist. In our center, blastocysts were simply shrunk by proper energy at first, then minor laser energy was used to slot the zona pellucida at the position of biopsy. The trophoblast cells to be biopsied were sucked into the biopsy needle through negative pressure, and then were cut by laser along the interface between zona pellucida and biopsy needle. Finally, trophoblast cells were completely segregated. Generally, the biopsied trophoblasts were lysed, and DNA was amplified by whole genome amplification (WGA, SurePlex, Illumina, Inc., San Diego, CA, USA) following the manufacturer’s protocol.

#### Blastocysts cryopreservation and thawing

We used vitrification technique for blastocyst cryopreservation, which was carried out using the Mukaida protocol with cryoloop [12]. To begin with, blastocysts were put into base medium containing 7.5% (v/v) DMSO and 7.5% (v/v) EG (vitrification solution I). After 2 min, the blastocysts were suspended in base medium containing 15% (v/v) DMSO and 15% (v/v) EG, 10 mg/ml Ficoll 70 (Pharmacia Biotech, Sweden, CB9248463) and 0.65 mol/l sucrose (vitrification solution II) for 30 s. Finally, they were put into liquid nitrogen as soon as possible.

Warming was performed in a four-well multidish using the Mukaida protocol. Briefly, blastocysts were incubated in base medium containing 0.33 mol/l sucrose (thawing solution I), base medium containing 0.2 mol/l sucrose (thawing solution II), and base medium at 37 °C for 2 min, 3 min, and 5 min.

Vitrified blastocysts were thawed on the morning of transfer day. After 30 min, hatching was assisted by laser with pulse length of 0.180 mS, applied for the thawed blastocysts. Only the expanded blastocysts were transferred after they were incubated for 4-5 h.

#### PGT technology

Before October 2016, PGT procedures were performed in the molecular laboratory of the center with a 24sure microarray chip (Illumina) according to the manufacturer’s protocol. Briefly, Cy3-labelled embryo WGA products were hybridized with Cy5-labelled SureRef (Illumina) reference male DNA WGA products. A microarray scanner (InnoScan 900) was used to scan microarray slides. And the images were analyzed using BlueFuse Multi software (BFM, Illumina). Aneuploid embryos that were unbalanced for the translocated chromosomes or/and abnormal for aneuploidy of chromosomes unrelated to the translocation were ascertained when those with a ratio greater than + 0.3 log 2 ratio were considered to be trisomic, and those with a ratio less than − 0.3 log 2 ratio were considered to be monosomic. Segmental abnormality in chromosomes unrelated to the parental rearrangement was indicated when chromosomal segmental aneuploidy of more than 10 Mb was involved.

After October 2016, NGS was implemented. After DNA fragmentation and library construction, the VeriSeq PGS kit—MiSeq (Illumina) was used for parallel sequencing and alignment, which were performed using the manufacturer’s protocol. The bioinformatic analysis was completed using BlueFuse Multi Software V4.4 (Illumina). Data quality, read depth, dynamic range/noise, the presence of ramping artifacts or step changes, and any recorded issues with biopsy or cell-to-tube transfer (e.g. the presence of lysed cells) were also taken into account in the final diagnosis.

As far as we know, NGS is expanding its applications in PGT given its capability to detect items of wide applications and detect unknown fragments. However, NGS also has limitations such as lower number of available bins of each chromosome compared to the a-CGH platform. Therefore, chromosome rearrangements that are predicted to be small and can be tested by a-CGH may not be detectable using NGS. By these means, we screen or diagnose numerical and structural chromosomal abnormalities from cells of trophectoderm. The embryos will not be thawed until the screening results come out.

#### Endometrial preparation and luteal phase support

In the control group, nearly all patients underwent fresh embryo transfer unless they had a tendency for ovarian hyperstimulation syndrome (OHSS) after controlled ovarian hyperstimulation (COH). Generally, fresh embryo transfer cases did not need extra endometrial preparation because the endometrium was able to reach its maximal thickness after COH. In our center, we usually use oral dydrogesterone (40 mg/d) and vaginal progesterone (200 mg/d) for each patient. Luteal support was initiated from the day of oocytes retrieval to the 12th week of pregnancy in most circumstances.

In the PGT group, all cases underwent frozen embryo transfer and the luteal phase support varied with the endometrial preparation protocol. At the third (or later) menstrual cycle after oocyte retrieval, natural ovulation was monitored by ultrasonography. Thirty milligrams of oral dydrogesterone were administered daily from the day of ovulation until the 12th week of pregnancy. If the natural ovulation cycle was cancelled due to anovulation or poor endometrial development, an artificial cycle was used for endometrial preparation. Luteal phase support was started when the endometrial thickness reached at least 7 mm and continued daily until 12 weeks of gestation with 40 mg daily of oral dydrogesterone and 200 mg daily of vaginal progesterone. Ovulation induction cycle was used if the endometrium was still responding poorly after natural ovulation and artificial cycles. Luteal phase support was initiated from the day of ovulation and the dosage was between the above two protocols.

### Outcome measures

In each cycle, pregnancy outcomes of the first transfer (including cryopreserved and fresh embryo transfers) were evaluated, including biochemical pregnancy, clinical pregnancy, ongoing pregnancy, live birth, miscarriage rates of the first transfer as well as the cumulative live birth rates. Biochemical pregnancy is defined as a hCG serum levels of more than 25 mIU per milliliter as measured at 12 days after embryo transfer. Clinical pregnancy refers to the signals of fetal heart beating in gestational sac assessed by ultrasonography during the 7th or 8th week. Ongoing pregnancy is regarded as the presence of a vital fetus with a positive heart beat at 11–12 weeks of gestation. Live birth is defined as the delivery of a live-born baby infant after at least 28 weeks of gestation. Miscarriage means termination of pregnancy before 28 weeks of gestation or a fetal weight lower than 1000 g [13].

We compared the differences of pregnancy outcomes between PGT and control group in order to find out if PGT could improve pregnancy outcomes of chromosomal inversion carriers. First, we compared factors which could potentially influence the pregnancy outcomes between the groups. These included maternal age, history of recurrent spontaneous abortion (RSA), duration of infertility, body mass index (BMI), hydrosalpinx, uterine leiomyoma, previous pelvic or intrauterine surgery and uterine abnormality. Since we found that the duration of infertility and the RSA rate in the PGT group were significantly different compared to the control group and maternal age has always been regarded as being of influence to pregnancy outcomes, logistic regression analysis was performed to determine if the factors besides the assisted reproduction method used would have any impact on pregnancy outcomes. According to the American Society for Reproductive Medicine’s criteria [14], RSA is defined as pregnancy loss for twice or more. BMI is calculated when kilograms of weight are divided by meters square of height. Hydrosalpinx is observed by ultrasonography of adnexa and/or salpingography. Uterine abnormalities included endometrial polyps, untreated intrauterine adhesions and uterine malformations.

In the PGT group, the aneuploid rate was compared between the pericentric and paracentric inversion subgroups, and between the male and female carrier subgroups.

### Statistical analysis

All analyses were carried out using the Statistical Package for Social Science (SPSS) software release 20.0. An independent t-test and Chi-square test (including Fisher’s exact test) were used to compare the clinical characteristics of patients from the PGT and the control groups. As for the pregnancy outcomes, Chi-square test and binary logistic regression analysis were used to observe the effects of those potential factors, respectively, on the different pregnancy outcomes. The significance level was set at *P*<0.05.

As such, this study was determined to be exempt from Institutional Review Board review.

## Results

A total of 136 cycles from the couples undergoing PGT and 52 cycles from the control group undergoing IVF or IVF/ICSI were analyzed. The detailed karyotype information of each inversion is shown in Supplementary Table 1. Among the 136 PGT cycles, 37 cycles (27.21%) meet the indication for IVF/ICSI. In the control group, 14 cycles (26.92%) underwent IVF/ICSI, which showed no significant difference (*P* = 0.969). As a result, the effect on pregnancy outcomes by sperm factor can be balanced. In the control group, the duration of infertility was significantly longer than that in the PGT group (*P*<0.05). There were 16 (11.8%) cycles in the PGT group that had a history of RSA, while the control group had none (*P*<0.05). Maternal age, BMI, intrauterine abnormality, hydrosalpinx, uterine myoma and previous surgery of pelvic or intrauterine cavity in the PGT group were not statistically different from the control group. Long GnRH agonist protocol was most commonly chosen in both groups, and only GnRH-antagonist protocol was more frequently used in control group compared to PGT group. Furthermore, Wu Q. et al. concluded that gonadotropin dosage is not associated with embryonic aneuploidy or live-birth rates in Chinese women [15]. Therefore, we considered COH protocol as a relatively slight factor that might influence pregnancy outcomes in our study. The pregnancy outcomes are also displayed in Table 1. The cumulative live birth rate of the PGT group was 49.3% which was not statistically different from that of the control group (50.0%, *P*>0.05). Besides, we also compared the rates of live birth, biochemical pregnancy, clinical pregnancy, ongoing pregnancy and miscarriage between the two groups. All results showed no statistically significant difference.

**Table 1:** Clinical data of patients who underwent PGT or not

	PGT group	Control group	t/x^**2**^	***P*** value
**No. of Cycles**	136	52	–	–
**Duration of Infertility (year)**	2.92 ± 3.25	4.29 ± 2.51	2.751	0.007
**Maternal Age**	31.23 ± 4.94	31.02 ± 4.78	−0.261	0.794
**BMI**	24.06 ± 3.66	23.53 ± 3.36	−0.907	0.366
**RSA**	16 (11.8%)	0 (0.0%)	–	0.007(Fisher’s exact test)
**Uterine Abnormality**	31 (22.8%)	10 (19.2%)	0.280	0.597
**Hydrosalpinx**	13 (9.6%)	7 (13.5%)	0.603	0.438
**Uterine Leiomyoma**	8 (5.9%)	3 (5.8%)	–	1.000(Fisher’s exact test)
**Previous Pelvic or Intrauterine Surgery**	53 (39.0%)	26 (50.0%)	1.878	0.171
**COH protocols**				
**Long GnRH agonist**	87 (64.0%)	30 (57.7%)	0.631	0.427
**Short GnRH agonist**	36 (26.5%)	8 (15.4%)	2.579	0.108
**GnRH-antagonist**	11 (8.1%)	13 (25.0%)	9.661	0.002
**other protocols**	2 (1.5%)	1 (1.9%)	–	1.000(Fisher’s exact test)
**No. of days of ovarian stimulation**	9.74 ± 2.20	9.88 ± 2.12	0.400	0.690
**Gonadotropin dose (IU)**	1880.19 ± 910.67	1797.36 ± 1051.31	−0.534	0.594
**Cumulative Live birth rate**	67 (49.3%)	26 (50.0%)	0.008	0.928
**First Transfer per cycle**				
**Live birth rate**	55 (40.4%)	20 (38.5%)	0.061	0.804
**Chemical pregnancy rate**	84 (61.8%)	33 (63.5%)	0.046	0.830
**Clinical pregnancy rate**	73 (53.7%)	27 (51.9%)	0.046	0.829
**Ongoing pregnancy rate**	70 (51.5%)	27 (51.9%)	0.003	0.956
**Miscarriage rate**	7 (8.3%)	3 (9.1%)	0.017	0.895

To eliminate the potential effects from the duration of infertility and RSA on pregnancy outcomes, a binary logistic regression was performed and that analysis finally concluded that PGT obviously did not improve pregnancy outcomes in chromosomal inversion carriers (Table 2).

**Table 2:** Logistic regression for patients who underwent PGT or not

	B	OR	P value	95%CI
**Cumulative Live birth rate**				
Undergo PGT	–	–	0.978	–
Maternal Age	−0.119	0.887	< 0.05	0.831–0.947
Duration of Infertility (year)	–	–	0.602	–
RSA	–	–	0.778	–
**Live birth rate of first transfer**				
Undergo PGT	–	–	0.763	–
Maternal Age	−0.092	0.912	< 0.05	0.854–0.973
Duration of Infertility (year)	–	–	0.301	–
RSA	–	–	0.976	–
**Biochemical pregnancy rate of first transfer**				
Undergo PGT	–	–	0.868	–
Maternal Age	−0.083	0.921	< 0.05	0.865–0.980
Duration of Infertility (year)	–	–	0.144	–
RSA	–	–	0.708	–
**Clinical pregnancy rate of first transfer**				
Undergo PGT	–	–	0.788	–
Maternal Age	−0.081	0.923	< 0.05	0.868–0.981
Duration of Infertility (year)	–	–	0.159	–
RSA	–	–	0.905	–
**Ongoing pregnancy rate of first transfer**				
Undergo PGT	–	–	0.996	–
Maternal Age	−0.085	0.918	< 0.05	0.863–0.977
Duration of Infertility (year)	–	–	0.274	–
RSA	–	–	0.613	–

The PGT group was divided into two subgroups according to the inversion type, the pericentric inversion subgroup (392 embryos tested) and the paracentric inversion subgroup (93 embryos tested). Euploidy rate in the subgroup of pericentric inversion carriers was 60.71% (238/392), which was not higher than that of the paracentric subgroup (50.54%, 47/93, *P* = 0.075). The inversion-relevant aneuploid embryos involve not only the chromosome with inversion of maternal or paternal origin, but also the other chromosomal errors, because we believe that the aneuploid embryos were affected by its maternal or paternal carriers as long as the abnormal chromosome included the same chromosome as the inverted one. Moreover, the de novo aneuploid embryos were unaffected by the concerned inversion, meaning that the abnormal chromosome(s) were irrelevant to parental inversion. The frequency of inversion-relevant aneuploid embryos was not significantly higher in the pericentric subgroup than in the paracentric subgroup (22.08% vs 13.04%, *P* = 0.256) (Table 3).

**Table 3:** Embryos and pregnancy outcomes of pericentric or paracentric inversion carriers

	Pericentric	Paracentric	t/x^**2**^	***P*** value
**Cycles**	106	30	–	–
**Maternal Age**	31.27 ± 4.86	31.07 ± 5.30	0.202	0.840
**Embryos tested**	392	93	–	–
**Inversion-relevant aneuploids**	34 (22.08%)	6 (13.04%)	1.807	0.179
**Euploids**	238 (60.71%)	47 (50.54%)	3.213	0.073
**Cycles with live birth**	55 (51.89%)	12 (40.0%)	1.322	0.250

Similarly, the PGT group was also divided into two subgroups according to the gender of inversion carrier: male carrier subgroup (255 embryos tested) and female carrier subgroup (230 embryos tested). The frequency of aneuploidy and inversion-relevant aneuploid embryos of female carrier subgroup was not significantly higher than that of the male carrier subgroup (Table 4).

**Table 4:** Embryos and pregnancy outcomes of female or male inversion carriers

	Female carrier	Male carrier	t/x^**2**^	***P*** value
**Cycles**	65	71	–	–
**Maternal Age**	31.5 ± 5.1	30.9 ± 4.8	0.660	0.510
**Embryos tested**	230	255	–	–
**Inversion-relevant aneuploids**	21 (20.8%)	19 (19.2%)	0.080	0.777
**Euploids**	129 (56.1%)	156 (61.2%)	1.293	0.256
**Cycles with live birth**	28 (43.1%)	39 (54.9%)	1.907	0.167

## Discussion

As suggested by previous studies, chromosomal inversion carriers whose phenotypic features did not vary with the chromosomal rearrangement may produce unbalanced gametes with an odd number of cross-overs during recombination, which can cause miscarriages, abnormalities and other adverse pregnancy outcomes. Since PGT has been proven beneficial in improving pregnancy outcomes of reciprocal translocation or Robertsonian translocation carriers, we aimed to examine if PGT has similar benefits for chromosomal inversion carriers. The previous studies on the effect of PGT on chromosomal inversion carriers were limited by their small sample size. A case-control study with adequate samples or large-scale RCT is lacking [16]. In addition, many previous studies of inversion focused on predictive value of recombination frequencies using sperm-FISH. Since these studies were only aimed at male inversion carriers, the aneuploidy embryo rates and pregnancy outcomes were not examined [5, 6]. Therefore, it remained unclear whether PGT should be recommended to improve pregnancy outcomes for inversion carriers during genetic counseling or not. To our best knowledge, this study is the first comparing the differences of pregnancy outcomes between inversion carriers with PGT and those without PGT. The inverted chromosome of the carriers in both groups involved most autosomes and sex chromosomes except chromosome 9.

This study showed that PGT didn’t improve pregnancy outcomes of chromosomal inversion carriers who needed in vitro assisted reproduction. To completely eliminate the potential effect of fresh blastocyst transfer, we excluded the fresh transfer cases and re-analyzed the clinical characteristics and pregnancy outcomes of the two groups. However, there still was no statistically significant difference between the two groups. Additionally, the article by Shi,Y. et al. concluded that live-birth rate did not differ significantly between fresh-embryo transfer and frozen-embryo transfer among ovulatory women with infertility [17]. Therefore, we involved not only cryopreserved embryo but also fresh blastocyst transfers in the control group. Besides, a recent randomized controlled trial by Madani T et al. found that four different types of endometrial preparation methods (natural cycle with spontaneous ovulation; natural cycle with human chorionic gonadotropin (hCG) for ovulation induction; hormone replacement cycle (HRC) and HRC with pre-treatment with GnRH-a) for FET cycles appear to be equally effective in terms of implantation, pregnancy, miscarriage and live birth rates in women with normal menstrual cycles [18]. Therefore, the effects of endometrial preparation on pregnancy outcomes in these two groups can be overlooked.

Our study suggested PGT should not be recommended to all inversion carriers. In certain circumstances, such as maternal advanced age or history of recurrent spontaneous abortion, PGT can be used as an effective method to prevent another adverse pregnancy outcome as far as possible. With an aneuploid rate up to 41.24% (200/485) in the PGT group, pregnancy outcomes were surprisingly not significantly improved by PGT. While the reasons behind this result are not completely clear, limitations of PGT technology or the physiological and psychological effects on patients caused by the high cost and pressure of PGT are suspected [19, 20]. The underlying mechanisms of the phenomenon that people with a normal karyotype are also likely to produce aneuploid embryos are unknown, and there is no direct evidence to prove that adverse pregnancy outcomes such as miscarriage or stillbirth will definitely occur after transferring an unbalanced embryo. Therefore, PGT would not provide significant benefits for most inversion carriers.

In our perspective, clinicians may advise inversion carriers without any indications of in vitro assisted reproduction to try to get pregnant naturally at first. If they have had a history of adverse pregnancy such as miscarriage with chromosomally abnormal embryos, PGT can be considered to prevent repeated abortions to the greatest extent. If the couple also has indications of IVF/ICSI such as ovulation, salpinx or sperm factors but has no indications of PGT, clinicians may give a suggestion that they can choose IVF/ICSI. Once they undergo pregnancy loss after the first transfer with chromosomally unbalanced embryos, the remaining cryopreserved embryos need to be tested using PGT technology before the next transfer.

Researchers used to reckon that it is less likely for paracentric inversion carriers to produce recombinant gametes than for pericentric inversion carriers and that most paracentric inversions were innocuous [4]. For example, a recent retrospective study with 57 paracentric and 94 pericentric inversion carriers showed that the percentage of normal/balanced blastocysts was significantly higher in paracentric carriers than pericentric carriers [21]. In this study, inconsistent with previous research, the aneuploid rates of pericentric inversion carriers were not higher than those of paracentric inversions with similar pregnancy outcomes. There may be a bias arisen from a gap between the population of the two subgroups on the basis of the low incidence of paracentric inversion. Since we believed that the aneuploid embryos were affected by its maternal or paternal carriers as long as the abnormal chromosome included the same chromosome as the inverted one, the inversion-relevant aneuploids involve not only the chromosome with inversion of maternal or paternal origin, but also the complex one including other chromosomal errors. And the de novo aneuploid embryos were unaffected by the concerned inversion, meaning that the abnormal chromosome(s) were irrelevant with the parental inversion. In this study, the aberrant chromosomes in most aneuploids were de novo aneuploids. Our observations showed that there were no statistically significantly differences in de novo aneuploid rates between pericentric and paracentric inversion carriers. As is well-known, the production of de novo aneuploids is closely related to maternal advanced age, and the interchromosomal effect is inconspicuous just as many studies indicated [7, 21]. Therefore, paracentric inversion cannot be considered less pernicious than pericentric inversion in genetic counseling. Just like a large review of 446 cases of paracentric inversions found, paracentric inversion carriers do not appear to be free of risks of abnormalities or abnormal progeny and caution is recommended when counseling [22]. More samples and data are needed to verify the difference of gametes and pregnancy outcome originated from these two inversion types.

Since there have been several studies assessing reciprocal translocation samples whose meiotic segregation might be affected by the carrier’s gender, we wonder if it would be similar for inversion carriers [23, 24]. A recent retrospective study found that for pericentric inversion carriers with an inverted segment accounting for more than 57% of the whole chromosome, the rate of unbalanced rearrangement was significantly higher for paternal than maternal inversion [21]. In our study, however, the result of both aneuploids rate and pregnancy outcome showed no significant differences between female carrier subgroup and male carrier subgroup. One limitation was that we were not able to create more groups for analysis due to lack of samples.

For inversion carriers, the risk for unbalanced recombination may vary with the affected chromosome and involved region, location of the breakpoints, or size of the inverted segment [9]. Aside from inadequate samples, this study was a retrospective case-control research and the two groups were not randomly assigned, leading to a grouping bias. For instance, patients with a history of RSA would tend to choose PGT rather than just IVF or ICSI, meaning that these patients probably possessed other unknown factors resulting in adverse pregnancy outcomes.

## Conclusion

Our results showed that PGT was not as prominently beneficial to improve pregnancy outcomes of chromosomal inversion carriers as we used to think. We anticipate that our findings will assist clinicians during genetic counseling to give their most appropriate advice and set proper expectations for patients.

## Supplementary information

**Additional file 1.**

## Data Availability

The datasets used and analysed during the current study are available from the corresponding author on reasonable request.

## Abbreviations

PGT: Preimplantation genetic testing
IVF: In vitro fertilization
ICSI: Intracytoplasmic sperm injection
ART: Assisted reproductive technology
COH: Controlled ovarian stimulation
AMH: Anti-Mullerian hormone
AFC: Antral follicle count
PCOS: Polycystic ovarian syndrome
GnRH: Gonadotropin-releasing hormone
hCG: Human chorionic gonadotropin
WGA: Whole genome amplification
NGS: Next generation sequencing
a-CGH: Array-comparative genome hybridization
OHSS: Ovarian hyperstimulation syndrome
RSA: Recurrent spontaneous abortion
BMI: Body mass index
HRC: Hormone replacement cycle
FET: Frozen embryo transfer
