# Supplementary material for: Clinical outcomes of Preimplantation genetic testing (PGT) application in couples with chromosomal inversion, a study in the Chinese Han population
Source: Reprod Biol Endocrinol. 2020 Aug 5;18:79. doi: 10.1186/s12958-020-00635-7 (PMC7405424; doi:10.1186/s12958-020-00635-7)
Supplement: Supplementary file 1 — Additional file 1. [file 12958_2020_635_MOESM1_ESM.docx]

**Supplementary Table 1**

| **PGT Group** | **Female Karyotype** | **Male Karyotype** |
| --- | --- | --- |
| 1 | 46,XX,inv(1)(p11q21) | 46,XY |
| 2 | 46,XX,inv(1)(p13q21) | 46,XY |
| 3 | 46,XX,inv(1)(p22q25) | 46,XY |
| 4 | 46,XX,inv(1)(p11q12) | 46,XY |
| 5 | 46,XX,inv(1)(p11q21) | 46,XY |
| 6 | 46,XX,inv(1)(p11q12) | 46,XY |
| 7 | 46,XX,inv(1)(p13q21) | 46,XY |
| 8 | 46,XX,inv(1)(p12q21) | 46,XY |
| 9 | 46,XX,inv(1)(p13q21) | 46,XY |
| 10 | 46,XX,inv(1)(p13q21) | 46,XY |
| 11 | 46,XX,inv(1)(p13q21) | 46,XY |
| 12 | 46,XX,inv(1)(p11q21) | 46,XY |
| 13 | 46,XX,inv(1)(p22q32) | 46,XY |
| 14 | 46,XX,inv(1)(p11q12) | 46,XY |
| 15 | 46,XX,inv(1)(q13q21) | 46,XY |
| 16 | 46,XX,inv(1)(p11q12) | 46,XY |
| 17 | 46,XX,inv(1)(p11q12) | 46,XY |
| 18 | 46,XX,inv(1)(p12q12) | 46,XY |
| 19 | 46,XX,inv(1)(p11q12) | 46,XY |
| 20 | 46,XX,inv(1)(p35q12) | 46,XY |
| 21 | 46,XX,inv(1)(p22.1p35) | 46,XY |
| 22 | 46,XX | 46,XY,inv(1)(p11q21) |
| 23 | 46,XX | 46,XY,inv(1)(p12q12) |
| 24 | 46,XX,inv(9)(p12q13) | 46,XY,inv(1)(p35q12) |
| 25 | 46,XX,inv(9)(p12q13) | 46,XY,inv(1)(p35q12) |
| 26 | 46,XX | 46,XY,inv(1)(p22q21) |
| 27 | 46,XX | 46,XY,inv(1)(p13q21) |
| 28 | 46,XX | 46,XY,inv(1)(p11q21) |
| 29 | 46,XX | 46,XY,inv(1)(p34q32) |
| 30 | 46,XX | 46,XY,inv(1)(p11q21) |
| 31 | 46,XX | 46,XY,inv(1)(p11q21) |
| 32 | 46,XX | 46,XY,inv(1)(p13q21) |
| 33 | 46,XX | 46,XY,inv(1)(p13q21) |
| 34 | 46,XX | 46,XY,inv(1)(p13q25) |
| 35 | 46,XX | 46,XY,inv(1)(p13q21) |
| 36 | 46,XX | 46,XY,inv(1)(p32q32) |
| 37 | 46,XX | 46,XY,inv(1)(p12q12) |
| 38 | 46,XX | 46,XY,inv(1)(p11q12) |
| 39 | 46,XX | 46,XY,inv(1)(p11q12) |
| 40 | 46,XX | 46,XY,inv(1)(q22q31) |
| 41 | 46,XX,inv(9)(p12q13) | 46,XY,inv(1)(p13q21) |
| 42 | 46,XX,inv(2)(p12q22)/46，XYqh- | 46,XY |
| 43 | 46,XX,inv(2)(p11q13) | 46,XY |
| 44 | 46,XX,inv(2)(p11.2q12) | 46,XY |
| 45 | 46,XX,inv(2)(p11q12) | 46,XY |
| 46 | 46,XX | 46,XY,inv(2)(p11.2q37) |
| 47 | 46,XX | 46,XY,inv(2)(p11q12) |
| 48 | 46,XX | 46,XY,inv(2)(p14q21) |
| 49 | 46,XX | 46,XY,inv(2)(p23q13) |
| 50 | 46,XX | 46,XY,inv(2)(p11.1q13) |
| 51 | 46,XX | 46,XY,inv(2)(p11q12) |
| 52 | 46,XX | 46,XY,inv(2)(p12q21) |
| 53 | 46,XX | 46,XY,inv(2)(p23q37) |
| 54 | 46,XX | 46,XY,inv(2)(p11q12) |
| 55 | 46,XX,inv(3)(p12q25.2) | 46,XY |
| 56 | 46,XX,inv(3)(p26q13) | 46,XY |
| 57 | 46,XX,inv(3)(q13.2q25) | 46,XY |
| 58 | 46,XX,inv(3)(q21q25) | 46,XY |
| 59 | 46,XX,inv(3)(q21q25) | 46,XY |
| 60 | 46,XX | 46,XY,inv(3)(p13q21) |
| 61 | 46,XX | 46,XY,inv(3)(p25q29) |
| 62 | 46,XX | 46,XY,inv(3)(p24q22),inv(9)(p12q13) |
| 63 | 46,XX,inv(4)(p26q13) | 46,XY |
| 64 | 46,XX | 46,XY,inv(4)(p13q21) |
| 65 | 46,XX,inv(5)(p12q32) | 46,XY |
| 66 | 46,XX,inv(5)(q13q33) | 46,XY |
| 67 | 46,XX,inv(5)(q13.1q33.1) | 46,XY |
| 68 | 46,XX,inv(5)(q13.1q33.1) | 46,XY |
| 69 | 46,XX | 46,XY,inv(5)(q13q35) |
| 70 | 46,XX | 46,XY,inv(5)(q22q32) |
| 71 | 46,XX | 46,XY,inv(5)(p13q13) |
| 72 | 46,XX,inv(6)(p12q21) | 46,XY |
| 73 | 46,XX,inv(6)(p12q22) | 46,XY |
| 74 | 46,XX,inv(6)(p21.3p22.2) | 46,XY |
| 75 | 46,XX,inv(7)(p13q22) | 46,XY |
| 76 | 46,XX,inv(8)(p21q22) | 46,XY |
| 77 | 46,XX | 46,XY,inv(8)(p23q24.1) |
| 78 | 46,XX | 46,XY,inv(8)(p23q24.1) |
| 79 | 46,XX | 46,XY,inv(8)(p11.22q23.3) |
| 80 | 46,XX,inv(10)(p13q22),9qh+ | 46,XY,13pstk+ |
| 81 | 46,XX,inv(10)(p13q22),9qh+ | 46,XY |
| 82 | 46,XX,inv(10)(q21.2q22.2) | 46，XYqh- |
| 83 | 46,XX,inv(10)(q11.2q24) | 46,XY |
| 84 | 46,XX,inv(10)(q21.3q22.2) | 46,XY |
| 85 | 46,XX | 46,XY,inv(10)(q11q23) |
| 86 | 46,XX | 46,XY,inv(10)(q11q23) |
| 87 | 46,XX | 46,XY,inv(10)(q21q22) |
| 88 | 46,XX,inv(11)（p14q12) | 46,XY |
| 89 | 46,XX,inv(11)（q12q22) | 46,XY |
| 90 | 46,XX | 46,XY,inv(11)（p15q13) |
| 91 | 46,XX | 46,XY,inv(11)（p15q12) |
| 92 | 46,XX | 46,XY,inv(11)（p14.3q22.2) |
| 93 | 46,XX | 46,XY,inv(11)(p12q11) |
| 94 | 46,XX,inv(12)(p13q15) | 46,XY |
| 95 | 46,XX,inv(12)(q12q21) | 46,XY |
| 96 | 46,XX | 46,XY,inv(12)(p12q13) |
| 97 | 46,XX | 46,XY,inv(12)(q13q24.3) |
| 98 | 46,XX | 46,XY,inv(12)(q13q24.3) |
| 99 | 46,XX | 46,XY,inv(12)(q12q24.2) |
| 100 | 46,XX,inv(13)(p11q14) | 46,XY |
| 101 | 46,XX,inv(13)(p11q13) | 46,XY,1qh+ |
| 102 | 46,XX,inv(13)(q22q34),16qh+ | 46,XY |
| 103 | 46,XX,inv(14)(q22q32.3) | 46,XY |
| 104 | 46,XX,inv(14)(q22q32.3) | 46,XY |
| 105 | 46,XX | 46,XY,inv(14)(p11q12) |
| 106 | 46,XX | 46,XY,inv(14)(p11q12) |
| 107 | 46,XX,inv(15)(q21q23) | 46,XY |
| 108 | 46,XX,inv(17),21s- | 46,XY |
| 109 | 46,XX,inv(17),21s- | 46,XY |
| 110 | 46,XX,inv(17)(p11p12) | 46,XY |
| 111 | 46,XX,inv(17)(p11p12) | 46,XY |
| 112 | 46,XX | 46,XY,inv(17)(p11.2q23) |
| 113 | 46,XX | 46,XY,inv(18)(p11q12) |
| 114 | 46,XX | 46,XY,inv(18)(p11q12) |
| 115 | 46,XX | 46,XY,inv(18)(p11.1q11) |
| 116 | 46,XX,inv(19)(p11q12) | 46,XY |
| 117 | 46,XX,inv(19)(p12q12) | 46,XY |
| 118 | 46,XX | 46,XY,inv(19)(p11q12) |
| 119 | 46,XX,1qh+ | 46,XY,inv(19)(p11q13) |
| 120 | 46,XX,inv(20)(p11q11) | 46,XY |
| 121 | 46,XX | 46,XY,inv(22)(p12q13) |
| 122 | 46,X,inv(X)(p22.3q22) | 46,XY |
| 123 | 46,X,inv(X)(q11q28) | 46,XY（Y≤G） |
| 124 | 46,X,inv(X)(q11q28) | 46,XY（Y≤G） |
| 125 | 46,XX | 46,X,inv(Y)(p11q12) |
| 126 | 46,XX | 46,X,inv(Y)(p11q12) |
| 127 | 46,XX | 46,X,inv(Y)(p11q12) |
| 128 | 46,XX | 46,X,inv(Y)(p11q12) |
| 129 | 46,XX | 47,XX,inv(Y)(p11q12) |
| 130 | 46,XX | 46,X,inv(Y)(p11q12) |
| 131 | 46,XX | 46,X,inv(Y)(p11.1q11.22) |
| 132 | 46,XX | 46,X,inv(Y)(p11q11) |
| 133 | 46,XX | 46,X,inv(Y)(p11q12) |
| 134 | 46,XX,inv(9)(p12q13),22ph+ | 46,X,inv(Y)(p11.2q11.2) |
| 135 | 46,XX | 46,X,inv(Y)(p11.2q11.2) |
| 136 | 46,XX,22ph+ | 46,X,inv(Y)(p11q12) |
| **Control Group** | **Female Karyotype** | **Male Karyotype** |
| 1 | 46,XX,inv(1)(q31q41） | 46,XY |
| 2 | 46,XX,inv(1)(p12q12) | 46,XY |
| 3 | 46,XX,inv(1)(p11q12) | 46,XY |
| 4 | 46,XX,inv(1)(p12q12) | 46,XY |
| 5 | 46,XX,inv(1)(p13q12) | 46,XY |
| 6 | 46,XX,inv(1)(q31q41) | 46,XY |
| 7 | 46,XX,inv(1)(p13q21) | 46,XY |
| 8 | 46,XX | 46,XY inv(1) |
| 9 | 46,XX | 46,XY,inv(1)(p34q32) |
| 10 | 46,XX | 46,XY,inv(1)(p13q31) |
| 11 | 46,XX,inv(2)(p11q12) | 46,XY |
| 12 | 46,XX,inv(2)(p11q13) | 46,XY |
| 13 | 46,XX,inv(2)(p11q14) | 46,XY |
| 14 | 46,XX | 46,XY,inv(2)(p11.2q12) |
| 15 | 46,XX | 46,XY,inv(3)（p11p26） |
| 16 | 46,XX,inv(5)（q14q34） | 46,XY |
| 17 | 46,XX,inv(5)(q31q35.1) | 46,XY |
| 18 | 46,XX,inv(6)(p23q21) | 46,XY |
| 19 | 46,XX | 46,XY,inv(6)(q16q25) |
| 20 | 46,XX | 46,XY,inv(6)(p11p23) |
| 21 | 46,XX | 46,XY,inv(6)(p22p21.2) |
| 22 | 46,XX | 46,XY,inv(6)(p12q12) |
| 23 | 46,XX | 46,XY,inv(7)(q31q34) |
| 24 | 46,XX,inv(8)(q22q24.3) | 46,XY,15ps+ |
| 25 | 46,XX | 46,XY,inv(8)(q11.2q13) |
| 26 | 46,XX | 46,XY,inv(8)(q22q24.3) |
| 27 | 46,XX,inv(10)(p13q23) | 46,XY,add(13)(p13) |
| 28 | 46,XX,22ps+ | 46,XY,inv(10)(q21q22) |
| 29 | 46,XX | 46,XY,inv(10)(q21q23),16qh+ |
| 30 | 46,XX | 46,XY,inv(10)(q21.2q22.3) |
| 31 | 46,XX,inv(11)(p14q14) | 46,XY |
| 32 | 46,XX,inv(12)(q12q21) | 46,XY |
| 33 | 46,XX,inv(17)(p11p12) | 46,XY |
| 34 | 46,XX,inv(17)(p11p12) | 46,XY |
| 35 | 46,XX | 46,XY,inv(17)(p11p12) |
| 36 | 46,XX,inv(20)(p11q13) | 46,XY |
| 37 | 46,XX,inv(21)(q21q22) | 46,XY |
| 38 | 46,XX,inv(22)(q12q13.2) | 46,XY,21ps+ |
| 39 | 46,XX,1qh+ | 46,X,inv(X)(p11q28) |
| 40 | 46,XX,1qh+ | 46,X,inv(X)(p11q28) |
| 41 | 46,XX | 46,X,inv(Y)(p11.2q11.2) |
| 42 | 46,XX | 46,X,inv(Y)(p11q12) |
| 43 | 46,XX | 46,X,inv(Y)(p11q12) |
| 44 | 46,XX | 46,X,inv(Y)(p11q12) |
| 45 | 46,XX | 46,X,inv(Y)(p11q12) |
| 46 | 46,XX,21pstk+ | 46,X,inv(Y)(p11q12) |
| 47 | 46,XX | 46,X,inv(Y)(p11q12) |
| 48 | 46,XX,1qh+ | 46,X,inv(Y)(p11q12） |
| 49 | 46,XX | 46,X,inv(Y)(p11q11.2) |
| 50 | 46,XX | 46,X,inv(Y)(p11q12) |
| 51 | 46,XX | 46,X,inv(Y)(p11.2q11.23) |
| 52 | 46,XX | 46,X,inv(Y)(p11q12) |

w
